# Place of Birth and Sleep Duration: Analysis of the National Health Interview Survey (NHIS)

**DOI:** 10.3390/ijerph14070738

**Published:** 2017-07-07

**Authors:** Valerie Newsome, Azizi Seixas, Juliet Iwelunmor, Ferdinand Zizi, Sanjeev Kothare, Girardin Jean-Louis

**Affiliations:** 1 Department of Population Health, New York University School of Medicine, New York, NY 10016, USA; azizi.seixas@nyumc.org (A.S.); ferdinand.zizi@nyumc.org (F.Z.); girardin.jean-louis@nyumc.org (G.J.-L.); 2 Department of Kinesiology and Community Health, University of Illinois, Champaign, IL 61801, USA; jiwez@illinois.edu; 3 Department of Neurology, School of Medicine, New York University, New York, NY 10016, USA; sanjeev.kothare@nyumc.org

**Keywords:** sleep duration, nativity, health, environment

## Abstract

While sleep disturbance has been related to a number of negative health outcomes, few studies have examined the relationship between place of birth and sleep duration among individuals living in the US. Data for 416,152 adult participants in the 2000–2013 National Health Interview Survey (NHIS), who provided self-reported hours of sleep and place of birth were examined. Associations were explored between healthy sleep (7–8 h), referenced to unhealthy sleep (<7 or >8 h), and place of birth using multivariate logistic regression analysis. The mean age of the sample was 47.4 ± 0.03 years; 56% were female. Of the respondents, 61.5% reported experiencing healthy sleep, 81.5% reported being born in the US and 18.5% were foreign-born adults. Descriptive statistics revealed that Indian Subcontinent-born respondents (71.7%) were more likely to report healthy sleep compared to US-born respondents (OR = 1.53, 95% CI = 1.37–1.71, *p* < 0.001), whereas African-born respondents (43.5%) were least likely to report healthy sleep (OR = 0.78, 95% CI = 0.70–0.87, *p* < 0.001). These findings suggest that place of birth should be considered in the assessment of risk factors for unhealthy sleep.

## 1. Introduction

Too many (>8) or too few (<7) hours of sleep (also known as unhealthy sleep) have been linked to increased risk for a number of major health issues, namely cardiovascular diseases [1,2,3]. Unhealthy sleep has been identified as a significant contributor to cardiovascular disease in numerous countries including the US [4,5]. According to data from the National Health and Nutrition Examination Survey (NHANES), about 37% of US adults report sleeping less than the recommended duration of 7–8 h per night [6]. Unhealthy sleep, along with its related adverse outcomes, represents a substantial public health burden with regard to mortality rates, quality of life, and health care costs in the US, but has only recently begun to receive increased attention in the fields of public health and clinical practice [7].

In an effort to address disparities in health, it seems prudent to analyze differences in sleep duration across racial and ethnic groups, as these sleep differences are likely related to disparities in other chronic diseases [8]. While studies of this kind are limited, evidence has emerged recently indicating differences in sleep duration among racial and ethnic minorities, suggesting a trend among ethnic minorities (particularly blacks) reporting fewer hours of sleep than Whites in the US [8,9,10,11]. Ethnic differences in sleep duration have also been reported in the Netherlands, where ethnic minorities (African-Surinamese, South Asian-Surinamese, Turkish, Moroccan, and Ghanaian) reported shorter sleep durations than ethnic-Dutch (European descent Dutch) [12]. To further examine racial and ethnic differences in sleep durations, Whinnery, Jackson, Rattanaumpawan & Grandner [13] examined differences based on place of birth and reported that short sleep (<7 h) was most common among Blacks born in the US and among non-US-born Blacks. However, compared with Whites, Latinos born in the US had shorter sleep if they were in professional/management and support services occupations, but experienced longer hours of sleep if they were in labor occupations, while non-US-born Latinos were significantly less likely to report short sleep than were Whites. Their study also showed within-group differences regardless of respondents’ nativity, suggesting a need for more research in these populations. These findings suggest that immigrant populations may experience unique differences in sleep, highlighting the influence of the biological, ecological, and social influences on patterns of sleep, which may also operate as mechanisms for increasing risk for other chronic diseases.

Given the dearth of published data in this important public health area, we analyzed National Health Interview Survey data from 2003 to 2013 to investigate associations between sleep duration and place of birth, while controlling for various potential confounders including demographics, behavioral risk factors, and chronic diseases. The main hypothesis tested in our in our multivariate analysis was that individuals would report differing amounts of sleep based on their place of birth.

## 2. Methods

The National Health Interview Survey (NHIS) is a yearly cross-sectional household interview survey conducted by the Centers for Disease Control and Prevention’s National Center for Health Statistics. The NHIS uses a multistage area probability design, stratification, and clustering to allow extrapolation to the US civilian non-institutionalized population. Details on sample design are reported elsewhere [14,15]. Trained personnel from the US Census Bureau collected data according to procedures specified by the National Center for Health Statistics in face-to-face interviews using computer-assisted personal interviewing. Respondents during the face-to-face interviews provided socio-demographic data (age, sex, race/ethnicity, average family income, and education), health risks (smoking status and alcohol intake), and physician diagnosed chronic conditions or diseases (hypertension, diabetes, coronary heart disease, cancer, kidney disease, stroke, and myocardial infarction).

Participants’ self-report of sleep duration was ascertained by the question: “On average, how many hours of sleep do you get in a 24-h period?” All participants were asked to respond to the question on place of birth. Other variables related to disease risk, such as body mass index (BMI) were derived from self-reported weights and heights, smoking status (never smoked vs. current/past smoker), and education (high school degree/equivalent or greater vs. less than high school), health insurance (yes or no), employment status (yes or no), psychological distress within the last 30 days based on the Kessler-6 scaling system [15]. The NHIS study was approved by the Ethics Review Board (ERB) of the Center for Disease Control and Prevention and the National Center for Health Statistics. The data are publicly available.

## 3. Statistical Analysis

Analyses were based on NHIS data obtained from 927,102 adult respondents from 2003 through 2013, providing self-reported average hours of sleep and place of birth. Since the NHIS dataset includes data from different samples using a multistage area probability sampling design, all analyses were conducted using complex survey analysis techniques to account for the weights, strata, and clusters in the National Health Interview Survey design as recommended by the Center for Disease Control. These weights represent a product of weights for corresponding units computed in each of the sampling stages. Once these data were harmonized and standardized across available years, additional processing was unnecessary. 

Descriptive analysis was used to ascertain sleep duration and place of birth across all available years of observation. We analyzed participant characteristics using chi-square for categorical variables and *t*-tests for normally distributed continuous data. Univariate associations of several socio-demographic (race, education, marital status, and poverty), health risk (smoking status, alcohol consumption, physical activity, and emotional distress), and medical conditions (prevalent hypertension, diabetes, stroke, heart attack, and heart disease) were ascertained to determine significant predictors of self-reported sleep duration. Weighted logistic regression models were performed to evaluate the effect of place of birth on the odds of reporting healthy sleep. The United States was selected as the referent country for each analysis because it accounted for the largest number of respondents in the dataset. Regression models adjusted for variables related to socio-demographic (race, education, marital status, and poverty), health risk (smoking status, alcohol consumption, physical activity, and emotional distress), and medical conditions (hypertension, diabetes, stroke, heart attack, and heart disease). All analyses were performed using Proc Survey logistic–SAS (SAS Institute, Cary, NC, USA).

## 4. Results

Of the 415,678 study participants (ages 18 years or older), 15.9% were foreign born. Table 1 summarizes the socio-demographic and health characteristics of the participants in the study based on their place of birth: foreign-born versus US-born. Foreign-born participants were more likely to be younger, less educated, earn below the federal poverty level, and sleep 7–8 h a day. US-born adults were more likely to be former smokers, current and former drinkers, obese, and more likely to have diabetes, hypertension, and coronary heart disease compared to foreign-born ones. There was no difference in the rate of adequate physical activity (>150 min/week) between foreign-born and US-born participants. The weighted prevalence of healthy sleep (7–8 h) was 62.5% overall; for foreign-born individuals, it was 66.1% and for US-born individuals, it was 61.7%. Table 2 shows results of the logistic regression analyses indicating estimates of healthy sleep (7–8 h) based on place of birth, with US-born participants as the referent group. Adjusted odds ratios were calculated with simultaneous adjustment for age, sex, race, education, alcohol, smoking, BMI, exercise, diabetes, coronary heart disease, and sleep duration. Overall, we observed a 19% greater likelihood of sleeping 7–8 h among foreign-born participants, compared with US-born participants (OR = 1.19 (95% CI = 1.15–1.23), *p* < 0.01).

In Table 3, we contrast the demographic and health risk characteristics of NHIS participants based on their place of birth. Individuals from Indian Subcontinent (71.2%) and Asia (68.5%) were most likely to report sleeping 7–8 h daily. Table 4 presents results of the stepwise logistic regression predicting adjusted odds ratios of sleeping 7–8 h based on place of birth. In the fully adjusted model, only individuals from Mexico, Indian Subcontinent, and Asia had a greater likelihood of sleeping 7–8 h. In contrast, participants from Africa had a significantly lower likelihood of sleeping 7–8 h (OR = 0.86 (95% CI = 0.73–1.02), *p* < 0.01).

## 5. Discussion

Findings from the current study make two significant contributions to the literature on sleep and health. First, we noted that place of birth was independently associated with healthy sleep duration (7–8 h) in the US. Second, the relationship between place of birth and sleep duration varied by region.

Though our results are consistent with previous studies [10,12,16] finding a relationship between place of birth and sleep duration (particularly sleep problems), the current study found that place of birth was a strong determinant of heathy sleep duration (7–8 h), adjusting for several socio-demographic and medical factors. Another important finding in our study was that duration of time in the US also correlated with sleep duration. Our data showed that as foreign-born individuals live longer in the US, the likelihood of healthy sleep decreased. These findings are consistent with the literature on acculturation and sleep among foreign-born individuals, a growing segment of the US population, whereby acculturation may exacerbate sleep problems or prevent individuals from experiencing adequate sleep [16,17]. A similar phenomenon, known as the “Latino Mortality Paradox” has been noted, particularly among Latino immigrants to the US, where they arrive in the US with better health than even the most well-to-do whites, but the longer they live in the US, the more their health declines [18]. Foreign-born individuals compared to US-born individuals, both currently residing in the US, have different sleep durations, which arguably may be attributed to biological, ecological, or psychosocial factors. These findings have clinical, scientific, and public health implications.

## 6. Biological Explanations

Differences in sleep duration based on place of birth might be explained by circadian rhythm differences among racial/ethnic groups. Recent findings indicate that circadian rhythm—behavioral, mental, and physiological changes that follow a 24-h cycle often in sync with light and darkness stimuli in one’s environment—is associated with genetic ancestry making the compelling case that circadian rhythms are innate [19]. Studies have found that individuals of African ancestry have shorter endogenous free-running circadian rhythms (Tau) which indicates: (1) earlier wake-sleep time; (2) larger phase advances and shorter phase delays; (3) higher susceptibility to sleep phase advance disorder (where individuals go to bed earlier and wake up earlier); (4) decreased ability to adjust to seasonal changes where light exposure is more variable especially in higher latitude regions like the US compared to regions closer to the equator; and (5) decreased ability to adjust to nighttime shift-work [20,21]. Therefore, we argue that since Blacks on average tend to work longer days and are more likely to be nighttime shift-workers/forced night owls they are therefore more likely to suffer from circadian misalignment which in turn affects their total sleep time [21].

Further evidence of racial/cultural differences in circadian rhythms have been observed as early as childhood and throughout the lifespan, as childhood napping, nocturnal sleep and adult sleep architecture between African Americans and Whites appear to differ [22,23,24,25,26,27]. Studies show that people of African ancestry have shorter circadian rhythm and are more susceptible to advance sleep phase circadian misalignment when they travel West [27]. This might explain their sleep problems living in the US. Second, recent findings indicating that individuals of African ancestry have lower amounts of slow-wave sleep compared to Whites also provides compelling evidence for our current findings. Since slow-wave sleep generally occurs during the rapid eye movement phase of sleep, lower amounts of slow-wave sleep may be attributed to restricted sleep time, shorter total sleep time [28].

## 7. Ecological Explanations

Overwhelming evidence suggests that ecological factors, such as how close one lives to the equator may influence circadian rhythm and sleep [28]. This might explain why participants from the Indian Subcontinent, Mexico, Central America, and Caribbean Islands; regions geographically close to the equator, were more likely to report healthy sleep durations. Individuals who live closer to the equator are exposed to natural sunlight more often than individuals who live further away and are therefore more likely to have well-synchronized circadian rhythms (endogenous circadian markers that are aligned with 24-h light-and-dark cycle) [21]. Additionally, greater exposure to natural sunlight promotes greater melatonin production, a hormone responsible for inducing sleep. Arguably, individuals from Mexico, Central American, the Caribbean, and the Indian subcontinent have aligned circadian rhythms and consequently may be less susceptible to circadian misalignment and sleep disturbances, such as insufficient sleep, even after migration. Perhaps their biological circadian rhythms are entrenched and thus protected against geographical re-location. Further studies are needed to investigate this issue more thoroughly.

Although circadian biology and environmental factors play a role in explaining differences in sleep duration, they do not fully explain our nuanced findings. For example, our findings indicate that after adjusting for all possible confounders only individuals from Mexico, Indian Subcontinent, and Southeast Asia reported significant hours of healthy sleep duration. Although, these regions are located near the equator, there may be other factors protecting them from sleep disturbances, poor sleep, and unhealthy sleep duration (too many (>8) or too few (<7) hours). Previous studies indicate that a host of psychosocial factors, such as acculturative stress, may influence sleep duration [10,16,17]. As individuals from Mexico, Indian Subcontinent, and Southeast Asia residing in the US traditionally live in their homogenous ethnic enclaves, they may have greater protection against acculturative stress than other immigrants residing in more heterogeneous neighborhoods, and thus more likely to get healthier sleep and less likely to have sleep problems. Having more acculturative stress coupled with advanced sleep phase circadian misalignment may increase the likelihood of sleep disruptions and inadequate sleep. As we did not specifically measure acculturation in this study, future studies should explore the role of acculturation and acculturative stress in foreign-born individuals in the US and its negative effect on their sleep duration.

## 8. Conclusions

This study adds to literature evidencing differences in sleep durations by comparing racial and ethnic minorities in the US, while enabling us to draw inferences about plausible biological, ecological, and psychosocial explanations. It is important to take these explanations into account when attempting to elucidate differences or disparities in sleep by ethnicity and by immigrant status in particular. Also of note are the potential lessons to be learned from immigrants who are more likely to get healthy sleep, even while dealing with the effects of acculturation and discrimination, both of which have been found to impact sleep duration negatively [16,29]. Research tends to frame relationships between ethnicity and health through a deficit model. Our findings present an opportunity to learn more about those psychosocial factors associated with ethnicity and immigration that may be protective in the US with respect to sleep. Biology and ecology aside, it is possible that these findings will facilitate a better understanding of the psychosocial aspects of sleep patterns among foreign-born individuals residing in the US.

While our study presents interesting findings, there are limitations worth mentioning. Our study is cross-sectional and cannot reveal causal or temporal links between place of birth and healthy sleep durations in the US. Another limitation lies in the possibility that systematic biases in self-reporting of sleep duration may affect our conclusions. Sleep was subjectively reported and foreign-born respondents may think it is more socially desirable to report more or less sleep duration and we were not able to adjust for these effects. We are also unable to determine when and where the problem with sleep duration originated; in their place of birth, or in the US. Another limitation is that the question on sleep duration only asks about total sleep in a 24-h period, but did not consider the importance of other factors such as quality of sleep, or whether the hours of sleep represent nocturnal sleep or the combination of nighttime sleep period and daytime naps [17]. Also, we did not have any detailed descriptions of neighborhood characteristics or home sleep environment of respondents that may influence sleep duration [30]. Finally, the results pertaining to length of time in the US may indicate that some of the difference that we see in sleep durations among foreign-born individuals may be related to issues associated with the acculturation process.

In summary, with the exception of African immigrants our findings demonstrate that foreign-born individuals residing in the US have favorable healthy sleep durations. However, the longer these individuals reside in the US, the more likely their sleep becomes compromised, suggesting that there may be aspects of their experience as immigrants in the US that play a role in their patterns of sleep duration and subsequent health outcomes.

## Figures and Tables

**Table 1:** Demography by foreign-born status.

	General	Foreign Born	US Born	*p*
N (%)	415,678	76,935 (15.9)	33,8743 (84.1)	0.01
Female (%)	233,038 (56)	41,895 (54.5)	190,906 (56.4)	0.01
Mean Age	47.4 ± 0.03	44.2 ± 0.02	48.1 ± 0.03	0.01
Mean Body Mass Index (BMI)	27.0 ± 0.01	26.4 ± 0.02	27.2 ± 0.01	0.01
Physical Activity Duration/Week (Mean ± SE)	162.9± 0.38	162.5 ± 0.01	163.0 ± 0.01	0.70
No High School Degree or Equivalent	65,568 (15.9)	25,084 (33.1)	40,440 (12.0)	0.01
Living Below Federal Poverty Level (%)	57,090 (16.5)	15,685 (24.62)	41,371 (14.7)	0.01
Never Drinkers	88,203 (22.3)	27,567 (37.9)	60,636 (19.3)	0.01
Former Drinkers	57,884 (14.6)	7975 (10.8)	49,909 (15.3)	
Current Drinkers	228,807 (63.2)	34,587 (51.3)	194,220 (65.5)	
Never Smokers	84,749 (20.5)	9690 (12.5)	75,059 (22.0)	0.01
Former Smokers	89,271 (21.8)	11,071 (12.5)	78,200 (23.1)	
Current Smokers	238,069 (57.7)	55,572 (72.5)	182,497(54.9)	
Normal Weight BMI (18.5–24.9)	147,518 (38.0)	29,436 (41.7)	118,082 (37.3)	0.01
Overweight BMI (25–29.9)	139,320 (35.8)	27,575 (38.5)	111,745 (35.2)	
Obese BMI (≥30)	103,705 (26.3)	14,642 (19.9)	89,063 (27.5)	
Healthy Sleep (7–8 h)	174,305 (62.5)	35,534 (66.1)	138,584 (61.7)	0.01
Abnormal Sleep (≤6 h and ≥9 h)	108,844 (38.47)	18,743 (34.53)	90,101 (39.40)	0.01
Short Sleep, ≤6 h (%)	83,825 (29.6)	14,709 (27.1)	69,043 (30.2)	0.01
Long Sleep, ≥9 h (%)	25,100 (8.9)	4034 (7.4)	21,058 (9.2)	0.01
Diabetes	35,675 (7.9)	6143(7.5))	29,532 (8.0)	0.01
Coronary Heart Disease (CHD)	19,166 (4.3)	2281 (2.7)	16,885 (4.6)	0.01
Hypertension	12,1497 (27.2)	16,528 (20.4)	104,969 (28.5)	0.01

**Table 2:** Logistic regression for foreign-born status and healthy sleep (7–8 h/day).

	Odds Ratio	95% Confidence Interval	*p*
Odds Ratio ^a^	1.23	1.21–1.26	0.01
Odds Ratio ^b^	1.19	1.15–1.23	0.01
Age 45–64 years (ref 18–44 years)	1.10	1.07–1.13	0.01
Age >65 years	1.16	1.11–1.21	0.01
Female Sex	0.92	0.90–0.94	0.83
Non-White/Minority	0.69	0.67–0.71	0.01
No High School Education	0.81	0.78–0.85	0.01
Former Alcohol Drinker	0.85	0.81–0.88	0.01
Current Alcohol Drinker	0.97	0.94–1.00	0.07
Former Smoker	1.43	1.38–1.48	0.01
Current Smoker	1.54	1.49–1.59	0.01
Exercise	1.00	0.99–1.02	0.75
Overweight (BMI = 25–29.99)	0.89	0.87–0.92	0.01
Obese (BMI ≥ 30)	0.74	0.72–0.76	0.01
Diabetes	0.89	0.85–0.93	0.01
Coronary Heart Disease	0.84	0.79–0.89	0.01
Hypertension	0.85	0.85–0.88	0.01

^a^ = Unadjusted model; ^b^ = Model adjusted for age, sex, race, education, alcohol, smoking, bmi exercise, diabetes, coronary heart disease, sleep duration.

**Table 3:** Healthy sleep and country of birth by demography.

	ALL	US	Mexico	South America	Europe	Russia	Africa	Middle East	Indian Subcont.	Asia	SE Asia	*p*
N (%)	416,152 (100)	338,743 (84.5)	43,115 (7.8)	5042 (1.0)	7123 (1.9)	1117 (0.3)	2459 (0.6)	1409 (0.4)	3392 (0.8)	4771 (1.0)	6401 (0.01)	0.01
Female (%)	231,586 (51.8)	190,906 (52.2)	23,468 (48.4)	2817 (52.1)	4100 (53.8)	650 (54.0)	1144 (44.2)	645 (46.4)	1437 (44.5)	2777 (56.0)	3642 (53.9)	0.01
Mean Age	47.4 ± 0.03	48.1 ± 0.03	42.7 ± 0.08	44.3 ± 0.22	51.9 ± 0.22	49.0 ± 0.59	39.7 ± 0.27	43.9 ± 0.44	38.9 ± 0.24	46.3 ± 0.26	45.7 ± 0.20	0.01
Mean (BMI)	27.3 ± 0.01	27.5 ± 0.01	27.6 ± 0.03	26.1 ± 0.07	26.1 ± 0.06	25.9 ± 0.17	26.3 ± 0.10	25.8 ± 0.13	24.5 ± 0.07	23.1 ± 0.06	24.1 ± 0.05	
Physical Activity (Mean ± SE)	162.9 ± 0.38	163.0 ± 0.41	169.1 ± 1.68	164.6 ± 3.67	165.7 ± 2.74	164.9 ± 8.62	154.4 ± 3.69	153.8 ± 5.22	131.4 ± 3.11	154.9 ± 3.12	157.7 ± 2.86	
No High School	65,322 (13.5)	40,440 (10.6)	21,417 (48.2)	766 (13.8)	805 (10.5)	94 (8.8)	197 (7.0)	169 (12.0)	187 (6.4)	475 (9.3)	772 (11.3)	0.01
Living Below FPL	56,853 (12.3)	41,371 (10.9)	11,290 (26.9)	752 (13.7)	600 (8.2)	193 (17.5)	434 (17.1)	248 (20.9)	409 (11.2)	795 (14.6)	761 (10.5)	0.01
Never Drinkers	87,862 (22.3)	60,636 (19.3)	16,248 (39.5)	142630.6)	1168 (18.1)	284 (27.9)	1109 (49.7)	562 (45.0)	1810 (59.8)	1970 (44.4)	2649 (43.9)	0.01
Former Drinkers	57,649 (14.6)	49,909 (15.3)	5052 (12.5)	464 (9.8)	718 (10.4)	97 (8.4)	178 (7.5)	97 (7.6)	179 (5.8)	402 (9.1)	553 (9.6)	
Current Drinkers	227,548 (63.1)	194,220 (65.5)	17,892 (48.0)	2701 (59.6)	4495 (71.6)	594 (63.6)	983 (42.8)	622 (47.3)	1202 (34.4)	2069 (46.5)	2770 (46.60	
Never Smokers	84,411 (20.5)	75,059 (22.0)	5221 (11.6)	635 (12.4)	1250 (17.3)	182 (17.4)	229 (9.0)	256 (18.3)	258 (6.3)	583 (12.4)	738 (11.8)	0.01
Former Smokers	88,717 (21.8)	78,200 (23.1)	5375 (12.6)	826 (16.5)	1923 (27.7)	200 (18.0)	241 (10.3)	249 (17.4)	280 (8.5)	617 (13.0)	806 (13.0)	
Current Smokers	236,902 (57.7)	182,497 (54.9)	32,231 (75.8)	3538 (71.1)	3877 (55.0)	722 (64.6)	1977 (80.7)	896 (64.3)	2835 (85.3)	3525 (74.6)	4804 (75.3)	
Normal Weight BMI (18.5–24.9)	146,623 (38.0)	118,082 (37.3)	12,871 (31.5)	2089 (42.8)	2979 (42.8)	473 (46.8)	970 (41.6)	591 (43.8)	1836 (56.1)	2977 (70.8)	3755 (61.8)	0.01
Overweight BMI (25–29.9)	138,631 (35.80	111,745 (35.2)	16,986 (42.4)	1863 (40.3)	2454 (37.9)	362 (36.3)	914 (40.2)	506 (39.4)	1080 (34.6)	999 (24.1)	1722 (29.7)	
Obese BMI (≥30)	103,322 (26.2)	89,063 (27.5)	10,468 (26.1)	808 (17.0)	1218 (19.2)	171 (16.9)	422 (18.1)	215 (16.8)	262 (9.2)	206 (5.0)	489 (8.5)	
Healthy Sleep (7–8 h)	173,207 (62.5)	138,584 (61.8)	19,816 (67.0)	2327 (66.7)	2924 (65.2)	496 (67.3)	1046 (55.9)	607 (63.7)	1944 (71.2)	2506 (68.5)	2957 (61.2)	0.01
Abnormal Sleep (≤6 h and ≥9 h)	108,360 (37.5)	90,101 (38.2)	10,005 (33.0)	1170 (33.3)	1678 (34.4)	258 (32.7)	806 (44.2)	363 (36.3)	769 (28.8)	1213 (31.5)	1997 (38.8)	

**Table 4:** Logistic regression for region of birth and healthy sleep (7–8 h/day).

Country/Region of Birth	OR ^a^ (Unadjusted)	Odds Ratio ^b^ (95% Confidence Interval)	Odds Ratio ^c^ (95% Confidence Interval)	Odds Ratio ^d^ (95% Confidence Interval)	*p*
United States (ref)	1.00	1.00	1.00	1.00	
Mexico, Central America, Caribbean Islands	1.26 (1.21–1.20)	1.39 (1.34–1.45)	1.19 (1.11–1.26)	1.18 (1.11–1.26)	0.01 *
South America	1.24 (1.13–1.36)	1.26 (1.15–1.39)	1.10 (0.95–1.27)	1.06 (0.91–1.23)	0.45
Europe	1.18 (1.10–1.27)	1.14 (1.06–1.230	1.12 (1.01–1.26)	1.09 (0.97–1.22)	0.26
Russia (and former USSR areas)	1.27 (1.07–1.52)	1.20 (1.01–1.43)	1.06 (0.79–1.43)	1.07 (0.79–1.44)	0.36
Africa	0.78 (0.70–0.87)	0.95 (0.85–1.06)	0.94 (0.81–1.10)	0.86 (0.73–1.02)	0.01 *
Middle East	1.09 (0.93–1.27)	1.04 (0.89–1.21)	0.99 (0.78–1.25)	0.96 (0.75–1.21)	0.08
Indian Subcontinent	1.53 (1.37–1.71)	1.98 (1.77–2.21)	2.01 (1.72–2.35)	1.86 (1.57–2.20)	0.01 *
Asia	1.35 (1.23–1.47)	1.58 (1.63–1.95)	1.71 (1.49–1.96)	1.49 (1.28–1.73)	0.01 *
SE Asia	0.98 (0.92–1.05)	1.31 (1.22–1.41)	1.31 (1.18–1.45)	1.19 (1.07–1.34)	0.13
Age 45–64 years (ref 18–44 years)		0.93 (0.91–0.95)	1.03 (1.00–1.07)	1.11 (1.07–1.14)	0.64
Age >65 years		0.92 (0.89–0.94)	1.07 (1.03–1.12)	1.20 (1.15–1.26)	0.01
Female Sex		0.97 (0.95–0.99)	0.96 (0.94–0.99)	0.93 (0.90–0.96)	0.01
Non-White/Minority (Non-Hispanic Blacks, Asians, Whites)		0.70 (0.68–0.72)	0.63 (0.60–0.66)	0.66 (0.63–0.69)	0.01
No High School Education		0.73 (0.71–0.75)	0.78 (0.74–0.83)	0.79 (0.75–0.84)	0.01
Former Alcohol Drinker			0.79 (0.75–0.83)	0.82 (0.77–0.86)	0.01
Current Alcohol Drinker			0.97 (0.94–1.01)	0.98 (0.94–1.02)	0.16
Former Smoker			1.38 (1.32–1.44)	1.43 (1.36–1.50)	0.10
Current Smoker			1.54 (1.49–1.61)	1.55 (1.48–1.61)	0.01
Exercise			1.02 (0.99–1.05)	1.00 (0.97–1.04)	0.84
Overweight (BMI = 25–29.99)				0.90 (0.87–0.93)	0.01
Obese (BMI ≥ 30)				0.73 (0.71–0.76)	0.01
Diabetes				0.91 (0.86–0.96)	0.01
Coronary Heart Disease				0.85 (0.79–0.92)	0.01
Hypertension				0.86 (0.83–0.89)	0.01

^a^ = Unadjusted model; ^b^ = Model adjusted for age, sex, race, education; ^c^ = Model adjusted for age, sex, race, education, alcohol, smoking, exercise; ^d^ = Model adjusted for age, sex, race, education, alcohol, smoking, BMI, exercise, diabetes, coronary heart disease, sleep duration. * *p* < 0.01.
